# Liver transplantation for hepatocellular carcinoma: Improving eligibility without compromising outcomes

**DOI:** 10.1016/j.amsu.2021.102552

**Published:** 2021-07-10

**Authors:** Abu Bakar Hafeez Bhatti, Ammal Imran Qureshi, Rizmi Tahir, Talal Almas, Atif Rana

**Affiliations:** a Department of Hepato-Pancreatico-Biliary Surgery and Liver Transplantation, Shifa International Hospital, Islamabad, Pakistan; b RCSI University of Medicine and Health Sciences, Dublin, Ireland; c Department of Interventional Radiology, Shifa International Hospital, Islamabad, Pakistan

**Keywords:** Hepatocellular carcinoma, Living donor liver transplant, Recurrence, Alpha fetoprotein

## Abstract

**Background:**

In the context of liver transplantation for hepatocellular carcinoma (HCC), traditional transplant criteria appear restrictive. The objective of the current study was to determine risk factors for recurrence and improve transplant eligibility in patients with HCC.

**Materials and methods:**

This was a retrospective study of patients who underwent living donor liver transplant (LDLT) for HCC (n = 219). Largest tumor diameter, tumor number, AFP and neutrophil to lymphocyte ratio were assessed. Multivariate analysis was performed to develop risk scores. The new model was compared with seven previously published transplant criteria using receiver operator curves.

**Results:**

Largest tumor size >3.7 cm [*HR:2.6, P* = *0.02*], and AFP > 600 ng/ml [*HR:4.7, P* = *0.001*] were independent predictors of recurrence. Patients with risk scores of 0, 1–3, 4–6 and 7–9 had recurrence rate of 5.9%, 12.5%, 25% and 58.4% respectively. When compared with Milan criteria, Metro ticket 2.0, AFP model and Samsung criteria; transplant eligibility increased by 31.5%, 22.9%, 8.7%, and 7% respectively. Recurrence rate with the current model was 16/199 (8%) (P < 0.0001) and was comparable with other transplant criteria (6.9–9.1%). On ROC analysis, only Milan criteria (AUC = 0.7, P = 0.001) and the current model (AUC = 0.66, P = 0.01) showed significance for recurrence. All patients with high risk scores within Milan criteria had recurred at 3 years (P = 0.03).

**Conclusions:**

Low AFP can be used to select patients for LDLT outside traditional criteria for HCC, with comparable recurrence rates.

## Highlights

• There is an unmet need to expand transplant pool for hepatocellular carcinoma.
• We developed a model, based on tumor size cutoff of 3.7 cm, any tumor number, and AFP cutoff of 600 ng/ml; and compared it with other transplant criteria.
• There was considerable expansion in transplant pool with comparable outcomes.

## 1 Introduction

Liver transplantation (LT) is an established treatment option in patients with liver cirrhosis and hepatocellular carcinoma (HCC) [1,2]. Milan criteria is considered the benchmark for LT in patients with HCC [3]. Although patients transplanted for HCC within Milan criteria have low recurrence rates, it has been criticized for not incorporating biological markers and for being too restrictive [4]. Newer criteria incorporating various biological markers with more liberal cutoffs on tumor size and number have been proposed [[5], [6], [7], [8]].

One particular approach has been development of flexible models using various combinations of tumor size, number and AFP levels [5,9]. The French-AFP model showed low recurrence rate with AFP < 1000 ng/ml for tumors within Milan criteria and with AFP < 100 ng/ml outside Milan criteria [5]. Similarly, variable tumor size, number and AFP level cutoffs were used in the Metro ticket 2.0 model proposed by the Milan group [9]. Recently, a model of recurrence based on preoperative and postoperative risk factors for recurrence was proposed, and was shown to be superior to Milan criteria in predicting recurrence [4]. A majority of these criteria have been derived in the setting of deceased donor liver transplantation (DDLT).

Living donor liver transplantation (LDLT) is a viable alternative to DDLT for HCC. Issues pertinent to DDLT include waiting list dropout, competition for donor organs and outcome comparison with non-HCC patients [10]. Since these are not relevant in LDLT, transplant centers can offer LT outside traditional transplant criteria.

As an LDLT center, we perform a substantial number of transplants for HCC outside traditional criteria. The objective of the current study was to determine pretransplant risk factors for recurrence and improve transplant eligibility in transplant candidates using biological and tumor related risk factors.

## 2 Methods

This was review of a prospectively maintained database of patients who underwent LDLT at our center. A total of 898 transplants were performed between April 2012 and December 2019. For this study, patients who underwent LDLT between April 2012 and June 2019, and had HCC confirmed on explant histopathology were considered. After exclusion of patients with major vascular invasion and who expired during hospital admission, 219 patients were included in the current study. The database include data pertinent to patient demographics, pretransplant treatment, operative and tumor related variables, and post transplant follow up.

### 2.1 Patient selection for liver transplantation

Details of patient and donor selection have been previously reported [11]. All donors were between 18 and 50 years of age, blood group compatible and related to the patient. Protocol for transplantation in patients with HCC have also been detailed elsewhere [12]. All patients were discussed in multi disciplinary team meeting and an ethical review committee before transplantation. Main portal vein tumor thrombus and extra-hepatic metastasis were absolute contraindications to transplantation. Patients who met UCSF criteria were offered upfront transplantation. In addition, patients with largest tumor diameter up to 10 cm and AFP <1000 ng/ml were also considered for LDLT. Patients with larger tumors (>10 cm), AFP >1000 ng/ml, and macrovascular invasion were routinely considered for down-staging. Patients with expected delay in LDLT (>6–8 weeks) were offered *trans*-arterial chemo-embolization (TACE), radio-frequency ablation (RFA)/microwave ablation (MWA)/percutaneous ethanol ablation (PEA) as bridge to transplantation.

### 2.2 Prognostic factors for recurrence

Well established preoperative markers for HCC recurrence include tumor size, number, AFP level, and neutrophil to lymphocyte ratio (NLR) [[4], [5], [9], [13], [14], [15], [16]]. We used receiver operator curves ROC) to determine cutoffs for these variables in our patients. Only tumor size 3.7 cm *(AUC = 0.68, P = 0.003)* and AFP level 600 ng/ml *(AUC = 0.7, P* = *0.001)* were significant for recurrence. Since prognostic impact of >3 tumor nodules and NLR >5 is well established, they were also included in univariate analysis [3,4]. Significant factors on univariate analysis were included in multivariate model. The hazard ratios of significant factors on multivariate analysis were rounded off to the nearest integer and assigned risk scores as previously described [4].

### 2.3 Statistical analysis

Mean +SD and median (range) were reported for continuous variables as appropriate. For categorical variables, Chi square and Fischer test was used. Recurrence free survival (RFS) was calculated by subtracting date of recurrence from date of transplant. Kaplan Meier curves were used to calculate recurrence free survival (RFS) and Log rank test was used to determine significance. A P value < 0.05 was considered statistically significant. A model for transplant eligibility was developed based on risk scores. This model was compared with 7 previously published transplant criteria for eligibility (number of eligible patients for transplantation), recurrence rate and estimated 5 year RFS. These criteria included Milan criteria, University of California San Francisco (UCSF) criteria, Metro ticket 2.0, French-AFP model, Asan criteria, Tokyo criteria, and Samsung criteria [3,5,8,9,[17], [18], [19]]. We did not consider models that used preoperative biopsy or post transplant variables for risk estimation [4,6,7,20]. Receiver operator curves were used to determine C statistic for individual transplant criteria. The study was approved by the hospital institutional review board and ethics committee (IRB # 134-954-2020).

The current paper has been formulated and reported in accordance with the STROCSS criteria [15].

## 3 Results

### 3.1 Patient and tumor characteristics

A total of 219 patients were included in the current study. Median AFP level was 14.9 (0.7–3632) ng/ml. Median follow up was 20.4 (3.24–81.6) months. Median largest tumor diameter was 2.8 (0.1–9) cm. Median number of tumor nodules was 2 (1–15). Table 1 demonstrates patient and tumor characteristics.

**Table 1:** Patient and tumor characteristics.

		Number (n = 219)	Percent
Gender	Male	184	84
Etiology	HCV	165	75.3
	HBV/HDV	36	16.4
	HBV/HCV	9	4.1
	Cryptogenic	5	2.3
	Others	4	1.8
MELD score	<10	26	11.9
	11–20	118	53.9
	21–30	67	30.6
	>31	8	3.6
Largest tumor diameter (cm)	<3.7 cm	143	65.3
Number of tumors	One	108	49.3
	Two	51	23.3
	three	16	7.3
	multiple	44	20.1
Grade	Well/moderate	152	69.4
	Poor	64	29.2
	N/A	3	1.4
Microvascular invasion	Present	73	33.3
AFP (ng/ml)	<600	199	90.8
Neutrophil to lymphocyte ratio	≤5	159	72.6
	>5	35	16
	unknown	25	11.4
Pre transplant treatment	Received	53	24.2

### 3.2 Predictors of recurrence

On receiver operator curve (ROC) analysis, tumor size 3.7 cm *(AUC = 0.68, P = 0.003)* and AFP level of 600 ng/ml*(AUC = 0.7, P* = *0.001)* were significant for recurrence. Multivariate analysis identified tumor size >3.7 cm [HR: 2.6 (1.1–6.2), P = 0.02] and AFP >600 ng/ml [4.7 (1.8–11.8), P = 0.001] as independent predictors of recurrence as shown in Table 2.

**Table 2:** Univariate and multivariate analysis for recurrence free survival.

	Univariate analysis		Multivariate analysis	
	HR (CI)	P value	HR (CI)	P value
Tumor size > 3.7 cm	3.48 (1.6–7.8)	0.001	2.5 (1.1–5.8)	0.03
Tumor number > 3	2.5 (1.07–6.1)	0.03	2.4 (0.9–6.3)	0.054
AFP > 600 ng/ml	7.6 (3.3–17.5)	<0.0001	5.6 (2.4–13.2)	<0.0001
Neutrophil to lymphocyte ratio	0.9 (0.3–2.7)	0.9	–	–

**Fig. 1:**
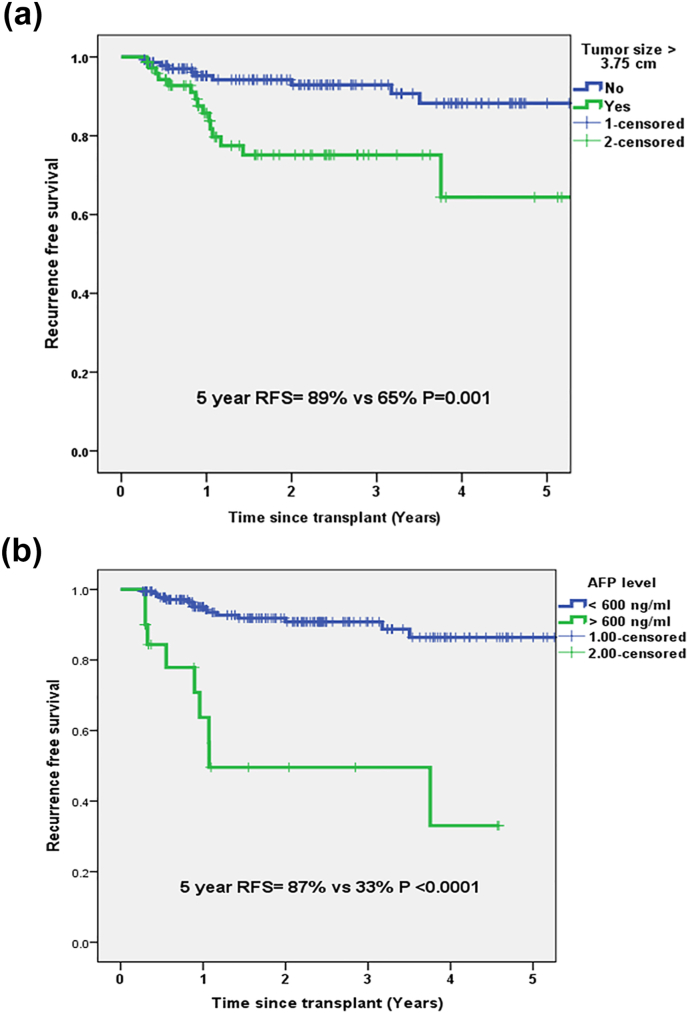
(a) Estimated 5 year RFS with largest tumor diameter cutoff 3.7 cm (b) Estimated 5 year RFS with AFP cutoff 600 ng/ml.

Estimated 5 year RFS with tumor size cutoff of 3.7 cm was 89% and 65% (P = 0.001) and AFP cutoff of 600 ng/ml was 87% and 33% (P < 0.0001) as shown in Fig. 1a and b. Both these factors were assigned risk scores as shown in Table 3.

**Table 3:** Assignment of risk score from hazard ratios.

	Hazard ratio	Risk score
Tumor diameter >3.7 cm	2.52	3
AFP >600 ng/ml	5.6	6

**Fig. 2:**
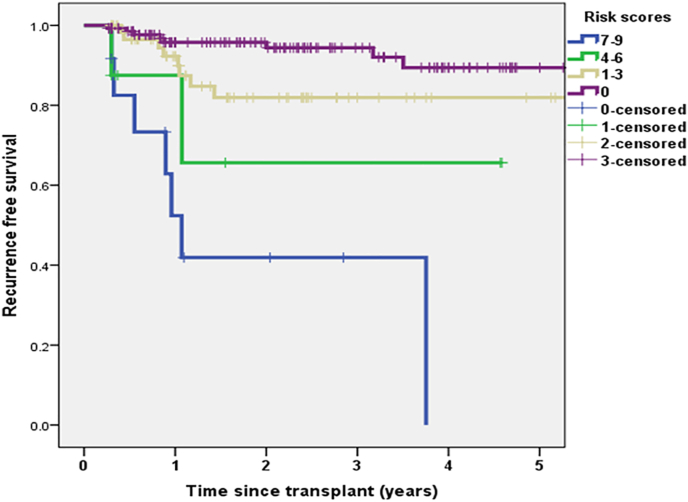
Estimated 5 year RFS based on risk scores.

The estimated 5 year RFS was 90% and 82% in low risk groups (risk score 0–3), 65% in intermediate risk (risk score 4–6), and 0 in high risk group (7–9) as all patients had recurred at 4 years (P < 0.0001) (Fig. 2).The actual recurrence rates based on risk scores is shown in Table 4.

**Table 4:** Recurrence rate in low, intermediate and high risk groups.

	Risk score	Number	Percent
Low risk (n = 199)	0	8/135	5.9
	1–3	8/64	12.5
Intermediate risk (n = 8)	4–6	2	25
High risk (n = 12)	7–9	7	58.4

### 3.3 Transplant eligibility and recurrence rates

**Fig. 3:**
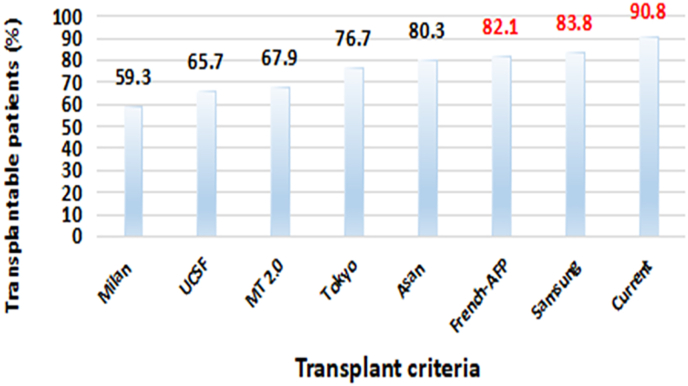
of transplantable patients meeting 7 different criteria based on our cohort of 219 patients.

The low risk group (risk score = 0–3) was considered transplant eligible. Overall transplant eligibility was 199/219 (90.8%). We used our patient cohort to assess transplant eligibility with 7 other transplant criteria as shown in Fig. 3. The current model including patients in low risk group allowed more patients to be transplanted when compared with all other criteria i.e. Milan = 31.5%, UCSF = 25.1%, Metro ticket 2.0 = 22.9%, Tokyo = 14.1%, Asan = 10.5%, French-AFP model = 8.7%, and Samsung criteria = 7% as shown in Fig. 3.

On ROC analysis, Only Milan criteria (AUC = 0.7, P = 0.001) and the current model (AUC = 0.66, P = 0.01) were significant for recurrence. The 5 year estimated RFS with the current model was 87% which was comparable to other transplant criteria as shown in Table 5. Moreover, the UCSF criteria, Asan criteria, Tokyo criteria and French-AFP model had no prognostic significance when applied to our patient cohort.

**Table 5:** Recurrence rates and estimated 5 year RFS in patients who met various transplant criteria.

	Recurrence rate		Estimated survival	
	Number	Percent	5 year RFS	aP value
Milan criteria	9/130	6.9	89	0.003
UCSF criteria	14/144	9.7	86	0.1
MT 2.0	12/146	8.2	88	0.01
Tokyo	17/168	10.1	84	0.17
Asan	18/176	10.2	83	0.18
French-AFP model	16/180	8.9	85	0.06
Samsung criteria	6/176	9.1	83	0.004
Current model	16/199	8	87	<0.0001

a P values for survival between patients who were within and outside individual criteria.

**Fig. 4:**
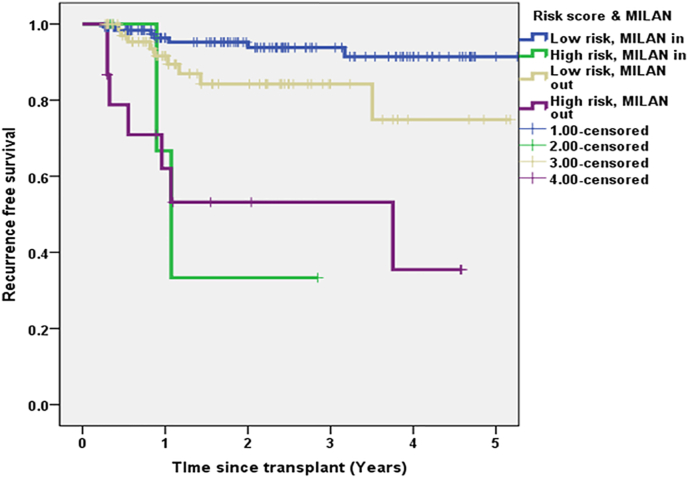
Estimated 5 year RFS within and outside Milan criteria based on risk scores.

We stratified patients who met Milan criteria based on risk scores. Estimated 5 year RFS in patients within and outside Milan criteria with low risk scores was 92% and 75% respectively (P = 0.034). Patients with high risk scores irrespective of Milan status had poor RFS as shown in Fig. 4.

## 4 Discussion

Milan criteria remains the benchmark for liver transplantation in HCC despite its limitations [3]. To increase transplant eligibility, more liberal cutoffs on tumor dimensions have been used with inclusion of indices of biological markers for better risk stratification.^5−9^In particular, flexible models involving judicious trade-offs between AFP levels and tumor dimensions have been proposed [5,9].

We compared the current model with 7 other transplant models with diverse selection criteria and mode of transplantation (DDLT vs LDLT). With the current model, more patients met eligibility criteria for transplantation, with a comparable RFS. In fact, transplant pool increased by more than 30% when compared to Milan criteria. We believe, this expansion was due to no cutoff of tumor number, and liberal expansion on tumor size if AFP was <600 ng/ml. The current model also allowed better risk stratification as patients with high risk scores had unacceptable recurrence rates even within Milan criteria.

An important aspect of the current study is risk stratification resulting in increased transplant eligibility with acceptable recurrence rates. Patients in the low risk group can be offered upfront transplantation. Patients with high risk scores should be referred for non-transplant treatment options owing to high risk of recurrence. A case for liver transplantation can be made if significant treatment response is observed in this group. For patients in the intermediate risk group, LDLT can be a viable treatment option. Due to lack of competition for donor organs and prerequisites to achieve outcomes comparable to non-HCC patients undergoing liver transplantation, long term survival of 50% is considered acceptable in LDLT [21,22].

This is because unique aspects of LDLT justify more liberal cutoffs in patient selection when compared with DDLT [22]. Alternatively, these patients can be down-staged and offered transplantation based on treatment response [[23], [24], [25]]. This also appears to be the group that can potentially benefit from adjuvant treatments or should be considered for experimental treatment protocols.

A similar model of risk scores has been proposed previously and was shown to be a better predictor of recurrence than Milan criteria [4]. However, its impact on transplant eligibility was not assessed. The pre-transplant model (Pre-MORAL) identified neutrophil to lymphocyte ratio (NLR), AFP and tumor size as independent predictors of recurrence. Indeed, NLR and PIVKAII are increasingly been recognized as markers for poor prognosis in HCC. However, their role as predictive factors after liver resection or liver transplant awaits more validation [[26], [27], [28], [29], [30], [31]]. We have previously shown that NLR was not a predictor of post transplant recurrence in our patients [12]. When compared with the Pre-MORAL model, current model showed improved risk stratification with more transplantable patients (48.9% vs 90.8%), with excellent long term survival (5 year RFS >80%).

Limitations of the current study include its retrospective design and assessment of various transplant criteria based on a highly selected patient cohort. Moreover we only looked at pre transplant AFP and various factors such as AFP response to downstaging were not evaluated.

## 5 Conclusion

AFP level before LDLT has prognostic impact on outcomes after liver transplantation. A low AFP level allows expansion on tumor size and number beyond traditional criteria with comparable outcomes. In the future, more refined risk stratification should allow more accurate identification of risk groups.

## Provenance and peer review

Not commissioned, externally peer-reviewed.

## Declaration of competing interest

None.

## Registration of research studies

1. Name of the registry: Research registry.

2. Unique Identifying number or registration ID: Researchregistry6274.

3. Hyperlink to your specific registration (must be publicly accessible and will be checked): https://www.researchregistry.com/browse-the-registry#home/registrationdetails/5fbb123a6827a8001b186b0b/
